# High-Resolution Computed Tomography: Lights and Shadows in Improving Care for SSc-ILD Patients

**DOI:** 10.3390/diagnostics11111960

**Published:** 2021-10-22

**Authors:** Barbara Ruaro, Elisa Baratella, Paola Confalonieri, Barbara Wade, Cristina Marrocchio, Pietro Geri, Annalisa Busca, Riccardo Pozzan, Alessia Giovanna Andrisano, Maria Assunta Cova, Marco Confalonieri, Francesco Salton

**Affiliations:** 1 Department of Pulmonology, University Hospital of Cattinara, 34149 Trieste, Italy; paola.confalonieri.24@gmail.com (P.C.); pietrogeri@gmail.com (P.G.); buscannal@yahoo.it (A.B.); riccardo.pozzan@outlook.it (R.P.); alessia.g.andrisano@gmail.com (A.G.A.); marco.confalonieri@asugi.sanita.fvg.it (M.C.); francesco.salton@gmail.com (F.S.); 2 Department of Radiology, Cattinara Hospital, University of Trieste, 34149 Trieste, Italy; elisa.baratella@gmail.com (E.B.); cristinamarrocchio@gmail.com (C.M.); m.cova@fmc.units.it (M.A.C.); 3 AOU City of Health and Science of Turin, Department of Science of Public Health and Pediatrics, University of Torino, 10124 Torino, Italy; barbarajenniferhellen.wade@unito.it

**Keywords:** systemic sclerosis-associated interstitial lung disease (SSc-ILD), interstitial lung disease (ILD), systemic sclerosis (SSc), high-resolution computed tomography (HRTC), pulmonary function tests (PFT), usual interstitial pneumonia (UIP)

## Abstract

The diagnosis and classification of systemic sclerosis-associated interstitial lung disease (SSc-ILD) is essential to improve the prognosis of systemic sclerosis (SSc) patients. The risk-stratification of disease severity and follow-up requires a multidisciplinary approach, integrating high-resolution computed tomography (HRTC) of the lung, pulmonary function tests (PFT), along with clinical and symptomatic evaluations. The use of HRCT in detecting SSc-ILD is not so much based on a definitive validation, but rather reflects the widespread clinician recognition of dissatisfaction with other modalities. However, due to the heterogeneity of SSc-ILD and the potential absence of symptoms in early or mild disease, it is prudent to consider as many parameters as possible in the assessment and monitoring of newly diagnosed patients. An early diagnosis meets the primary goal, i.e., the prevention of disease progression. The current first line treatment regimens are mainly centered on immunosuppressive therapy. This review assesses the role HRCT plays in optimizing care and improving clinical outcomes in SSc-ILD patients.

## 1. Introduction

Systemic sclerosis (SSc) is an autoimmune disease characterized by vasculopathy and fibrosis of the skin and multiple internal organs. The pathogenesis of SSc is complex and not yet fully understood, as environmental and genetic factors are involved, leaving the mechanisms and aetiology still to be unravelled [1,2,3,4]. A dysregulated immune response to unknown triggers in genetically predisposed subjects may lead to endothelial damage. The endothelial cells, which are aberrantly activated or damaged, favour the development of structural vascular changes such as destructive vasculopathy, proliferative obliterative vasculopathy and/or tissue fibrosis [1,2,3,4,5,6,7]. Furthermore, inflammatory cells activate SSc fibroblasts and modify the extracellular matrix metabolism through soluble factors and the development of autoantibodies. Moreover, the SSc fibroblasts also acquire the ability to selectively respond to pro-fibrotic cytokines and growth factors, persistently producing excessive amounts of extracellular matrix. Thus, various types of individually activated cells interact with one other and co-ordinately cause damage at the levels of the different organs, such as in the lung [8,9,10]. In this scenario, although major advances have been made in the identification and prognostic evaluation of SSc-ILD, the pathogenesis of interstitial lung disease (ILD) in systemic sclerosis (SSc-ILD) remains an open question and accurate epidemiological risk factors are still scanty [1,2,3,4]. However, there is a high prevalence of lung involvement in SSc early diagnosis, making for the necessity of a tailored approach which takes into consideration the patient’s probability of progressive disease, due to its considerable impact on prognosis [2,3]. Indeed, despite recent advances in treatment, 40–75% of SSc patients have reduced pulmonary function and ILD, which is usually observed within the first 3 years from diagnosis and is currently the major cause of death in SSc patients (70–90%) [3,4,5,6,7,8,9,10]. The European Scleroderma Trials and Research group (EUSTAR) studied 3,656 SSc patients, reporting that 53% of diffuse cutaneous SSc cases had ILD, as did 35% of cases with limited cutaneous SSc [11,12,13]. A cumulative 5-year survival rate for SSc patients from diagnosis of 84.1% and 74.9% at 10 years has been reported, respectively [4,5,6,7]. SSc-ILD tends to occur with increased severity and/or increased progression risk in patients with Scl-70 (anti-topoisomerase I) antibodies, for the male gender and those of African–American ethnicity [13,14,15]. SSc-ILD is most often associated with a histological pattern of nonspecific interstitial pneumonia (NSIP), whilst usual interstitial pneumonia (UIP) and other histologic patterns are less commonly observed [16,17,18,19,20]. However, differences in histological patterns do not appear to have prognostic significance in SSc-ILD [16,17,18,19,20,21].

Considering the heterogeneity of SSc-ILD and the potential absence of symptoms, patients may be diagnosed earlier and more efficiently through a multidisciplinary evaluation and team collaboration [22,23,24,25,26,27,28,29]. Furthermore, it is prudent to consider as many parameters as possible to enhance clinical outcomes when assessing and monitoring newly diagnosed patients. Indeed, the advent of high-resolution computed tomography (HRTC) has revolutionised the routine detection and management of ILD in SSc, making it the current gold standard for SSc-ILD diagnosis [30,31,32,33,34,35,36]. This review explores the role of this imaging technique in the identification, staging and follow-up of ILD in SSc patients.

## 2. The Role of HRTC in SSc-ILD

Before the advent of Computed Tomography scanning (CT), the identification of ILD was often a difficult task, even if early autopsy studies reported that up to 100% of patients had parenchymal involvement. Indeed, as many as 90% of SSc-ILD patients have interstitial abnormalities on high-resolution computed tomography (HRCT) and 40–75% have changes in pulmonary function tests (PFT) [19,20,21,22]. The clinical history of these patients usually reveals the insidious onset of exertional dyspnoea and a nonproductive cough. A clinical evaluation, with lung auscultation, usually reveals bilateral basilar dry inspiratory crackles. Frequently SSc-ILD patients are positive for SSc-specific antibodies, anti-topoisomerase I (anti-Topo I) and anti-Th/To and, less commonly, anti-centromere antibodies (ACA) [14,15]. Plain chest radiography is much less sensitive in the detection of lung involvement than CT and provides a poor evaluation of the extent of interstitial disease [36,37,38,39]. PFT are seriously limited by the wide range (80–120%) of normal values, which are calculated on the basis of the age, gender and height of the patient [36,37,38,39]. Although bronchoalveolar lavage (BAL) is not a suitable routine screening test, a BAL neutrophilia is more often present in SSc when HRCT reports more extensive disease [10,13]. However, the association of BAL abnormalities with clinically significant lung disease still remains to be clarified and, although symptoms, chest radiography, PFT and BAL findings may be striking in severe lung disease, at times they do not suffice for diagnosis in early lung disease [11,12,13].

The use of HRCT in the detection of ILD expresses the widespread clinical recognition of dissatisfaction with other modalities, rather than being based on definitive validation. That is to say, the last two decades have proven HRCT to be clinically useful in the detection of ILD; therefore, it has consequently assumed a pivotal role by default. Indeed, several studies have addressed the potential of lung HRCT as a screening tool for ILD in SSc [16,35,36,37,38,39,40,41,42,43,44,45]. However, radiation exposure remains a question to be taken into consideration. The most recent guidelines recommend that the radiation dose for the inspiratory volumetric acquisition should be around 1–3 mSv [46]. Launay et al. [16] made a detailed assessment of 90 consecutive SSc patients who had had two HRCTs and reported that 40/90 had normal findings at the first HRCT as did 34/40 at the second follow-up HRCT.

Moreover, the data also indicated two subsets of SSc patients: one with very low risk for IL D development and the other where ILD was present and required further surveillance. This observation was supported by data from a prospective Norwegian SSc cohort study, where serial lung HRCT and concurrent PFT were analysed in 305 consecutive, unselected patients [37]. In this study a multivariate outcome analyses demonstrated that normal diffusion lung capacity (DLCO) at baseline was associated with an absence of lung fibrosis at follow-up. A longitudinal analysis showed no statistically significant differences in the average annual fibrosis progression rate or average total FVC decline.

As interstitial lung disease is a major mortality factor in SSc, the routine use of a test can be justified as long as it establishes a low intermediate term probability of this complication and allows for less frequent pulmonary function monitoring [37]. However, a recommendation that routine baseline thoracic HRCT should be performed in SSc has not yet been made by expert groups [8,13,25,39]. In 2019, ILD was prospectively assessed in Norwegian SSc patients (Nor-SSc) in a nationwide cohort study on 815 SSc residents in Norway from 2000 to 2012 [39]. A total of 650 lung HRCT scans were available for fibrosis quantification at baseline (80%) and follow-up and 703 pulmonary function tests were assessed at baseline (86%) and follow-up. Vital status and standardized mortality ratios (SMR) were estimated at the end of the study (2018) in the 630 incident Nor-SSc cases and 15 individually matched control subjects, and cumulative survival rates were computed. At baseline, 324 of the SSc subjects (50%) had HRCT documented ILD and 46% had pulmonary function declines, consistent with ILD progression. There was a statistically significant correlation between the mortality rate and extent of the lung fibrosis, which was demonstrated by an SMR increase from 2.2 with no fibrosis, to 8.0 with more than 25% fibrosis. There was an inverse correlation between the SMR value and the FVC baseline percentage, which increased at all FVC levels below 100%. The 5 and 10-year survival rates correlated with the presence/absence of lung fibrosis in subjects with normal-range baseline FVC (80–100%), which were 83% and 80%, respectively, with no fibrosis and 69% and 56%, respectively, with lung fibrosis (*p*-value = 0.03) [39].

## 3. HRTC vs. Pulmonary Function Tests

**Figure 1:**
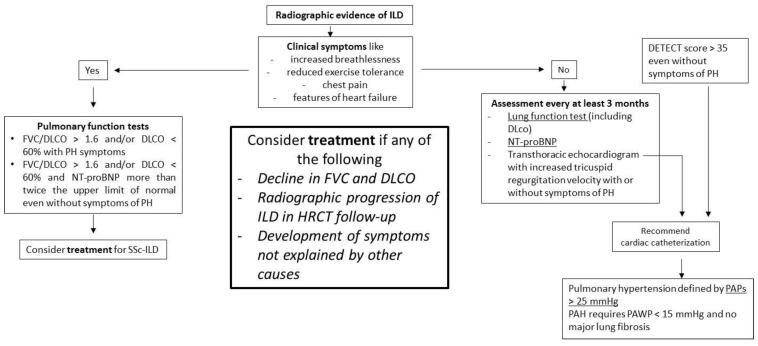
Practical algorithm about the use of HRCT/PFT in SSc ILD.

Considering the aforementioned limitations of HRCT and pulmonary function tests (PFT), several authors reported that the combined use of these tests is able to provide more informative and reliable prognostic information than when either is used alone [35,43]. Indeed, we are of the opinion that HRCT should be used to assess the presence of ILD as well in the assessment and quantification of dyspnea and cough, and that PFT should be performed in newly diagnosed SSc patients with appropriate follow-up investigations in ILD patients, which could include FVC, DLCO and HRCT assessments. Moreover, in line with other authors, to increase the possibility of an early diagnosis we propose yearly PFT with a low threshold for SSc patients with no known ILD and recommend the use of HRCT if there is a decline in FVC and/or DLCO and/or a change in the clinical scenario (e.g., increased dyspnea or audible crackles on chest auscultation) [42] (Figure 1).

PFT usually reveals a restrictive pattern with reduced forced expiratory volume in 1 s (FEV1) and FVC, mainly due to parenchymal involvement, along with a normal or slightly increased FEV1/FVC ratio. Restrictive lung disease due to severe thoracic cutaneous involvement has also been reported. Diffusing capacity of the lung for DLCO is the most important functional test as it not only assesses the interstitial space between the alveolar and endothelial surfaces, but also the integrity of the lung vascular bed. Indeed, there may be a reduction DLCO in either parenchymal fibrosis or vascular abnormalities of pulmonary hypertension. Moreover, recently a rapid DLCO decline has been identified as the single most significant marker of poor outcome [21,24,25]. Furthermore, it has been shown that DLCO provides the best estimate of the extent of ILD on HRCT [21,25,42]. A recent systematic review of 219 SSc-ILD studies reported that the predicted FVC% was the primary endpoint in 70% of the studies, and that DLCO assessment was the primary endpoint in 11% [45,46]. Data from the Pittsburgh Scleroderma Databank demonstrated that the highest declines in FVC often occur early, i.e., within the first 3 years of SSc onset, even in asymptomatic patients [45,46,47]. This was further supported by the results of the Scleroderma Lung Study (SLS) I study [48], where an association between the fibrosis extent, assessed by HRCT at baseline and an FVC decline, were reported as being more evident during the first 2 years after disease onset [48]. In our experience and in line with literature data, a decline from baseline of 5–10% in FVC and 10–15% in DLCO in an SSc-ILD patient should be further evaluated, as it may be indicative of disease progression, even if absolute values remain above the 80% threshold [49]. Goh et al. reported that a decline of ≥10% in FVC, or a 5–9% decline in FVC, together with a ≥15% in DLCO decline, was associated with a higher risk of subsequent mortality in SSc-ILD patients [50], attributed to disease progression in patients with extensive lung fibrosis. Man et al. reported that patients with a longer disease duration (>4 years) are less likely to have a future decline in lung function than subjects with early-stage disease [51].

It has also been recommended that these values be taken into consideration during ILD assessment, as both FVC and DLCO can be influenced by SSc disease processes other than ILD [45]. Other authors reported that FVC correlates poorly with the quantitative extent of fibrosis, whilst DLCO is the best predictor of HRCT-measured ILD [52], although the reliability of lung function as a predictor of SSc-ILD remains controversial. A recent study on 256 SSc patients extrapolated from patients with radiographic ILD (188 pts; 71%), 59 patients (31%) had a normal FVC (predicted ≥80%). Accurate DLCO measurements were available for 151/256 patients, 65/151 (43%) had a DLCO of ≥60% predicted [53]. These data highlight the limitations of PFT, showing that normal FVC and DLCO measurements in SSc patients do not exclude the presence of ILD nor do they obviate the need to pursue HRCT imaging to definitively assess for ILD. Due to the heterogeneity of technical factors, diurnal or seasonal changes and patient-related factors, PFT alone does not suffice to determine the true extent of ILD, and changes in PFT parameters do not consistently mirror changes in the extent of radiographic fibrosis [53].

## 4. HRCT Acquisition

According to the most recent guidelines, high-quality images are required to assess patients with known or suspected interstitial lung diseases, and a volumetric CT scan is preferred to a sequential CT to improve the characterization of the disease [46]. Indeed, a volumetric acquisition provides fine-detailed images and multiplanar reconstructions that help not only in a precise assessment of the distribution and extent of the disease, but also in the identification of additional findings, facilitating comparisons at follow-up. Movement-related artifacts during HRCT acquisition can be avoided by using the thinnest collimation, highest pitch and shortest rotation time. Although the use of an “ultralow-dose CT protocol” is not generally advocated for the assessment of interstitial lung diseases, when acquiring the CT scan, it is essential to reduce the delivered dose to a minimum, especially in SSc patients as they may require repeated follow-ups. Therefore, the tube potential and current, generally set at 120 kVp and ≤240 mAs, should be tailored to the patient’s constitution, reducing the kilovoltage and milliamperage for lower weight patients, e.g., to values of 100 kVp, along with the adoption of techniques that avoid unnecessary exposure, e.g., tube current modulation.

**Figure 2:**
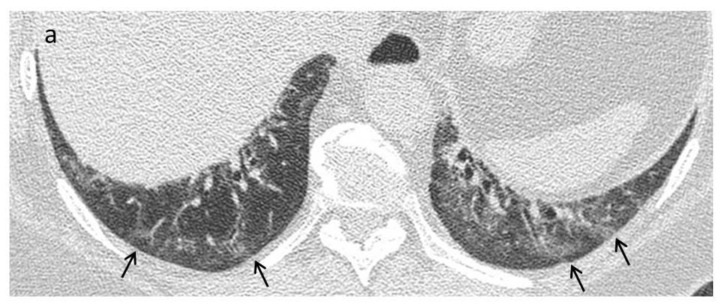

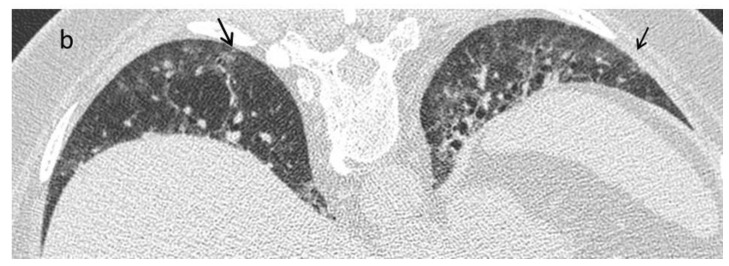
Early stage. A 42-year-old female with a diagnosis of SSc. (**a**) Axial High Resolution CT image shows subtle ground glass opacities (black arrows) in the peripheral areas of lung bases. An additional scan with patient in prone position can be helpful to differentiate gravity-related interstitial alterations from disease-related alterations. (**b**) The persistence of ground-glass opacities in the prone position is related to a nonspecific interstitial pneumonia (NSIP) pattern.

The patient should be supine for the first volumetric scan. Care must be taken to scan at the end of full inspiration as an inadequate inspiration increases lung attenuation and generates false images, such as ground-glass opacities (GGO) and fine reticulations, which may lead to misinterpretations [46,54]. However, if subtle ground-glass opacities or fine reticulations are present on supine images, a second acquisition, with the patient in a prone position, can be acquired to differentiate gravity-related interstitial alterations in dependent areas of the lung from disease-related alterations (Figure 2). As systemic sclerosis typically affects the posterior and basal portions of the lungs, this is of particular importance [46,54,55,56]. This acquisition can be volumetric or sequential and can be limited to the lower lobes [56]. Contiguous or overlapping thin section (≤1.5 mm) high-spatial resolution reconstructions of the acquired images are essential.

## 5. HRCT Pattern in SSc-ILD

**Figure 3:**
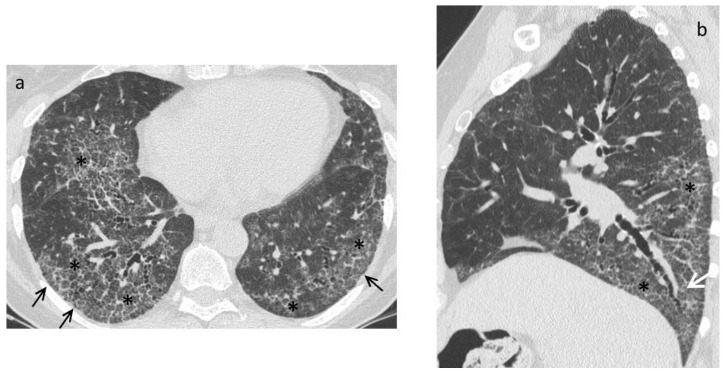
Late stage. High resolution CT images in axial (**a**) and sagittal (**b**) plane show a fibrotic nonspecific interstitial pneumonia (NSIP) pattern characterized by the presence of bilateral extensive ground glass opacities (*) with a peripheral and symmetric distribution with signs of fibrosis due to the presence of reticulations (black arrows) and traction bronchiectasis (white arrows).

**Figure 4:**
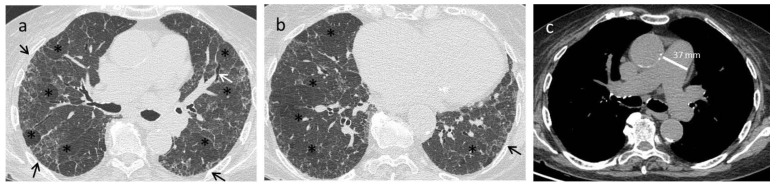
Pulmonary Hypertension. An 82-year-old female with a diagnosis of SSc. Axial High Resolution CT images show extensive subpleural reticulations (black arrows) and traction bronchiectasis/bronchiolectasis (white arrows), compatible with a UIP probable pattern (**a**,**b**). Signs of pulmonary hypertension, a common complication of this disease, can also be appreciated. On high resolution CT scan there is diffuse mosaic attenuation (*) with enlarged peripheral vessels (**a**,**b**), and a dilatation of the main pulmonary artery can be recognized using the mediastinal window setting (**c**).

A systematic approach is essential when evaluating an HRCT for systemic sclerosis to ensure a thorough assessment of the entire thorax. First, each finding must be correctly defined when reporting the alterations observed in the lungs, using the definitions provided in the glossary of terms for thoracic imaging by the Fleischner Society as references as reference [57]. The most frequent HRCT early-stage pattern is nonspecific interstitial pneumonia (NSIP), with a greater proportion of GGO and a lower proportion of coarse reticulation (Figure 3). A report on an HRCT series of 216 patients showed that 50% of the observed abnormalities were GGO, which was generally prominent even when reticular changes were more extensive [57,58,59,60,61]. However, a usual interstitial pneumonia (UIP) pattern, characterised by honeycombing and traction bronchiectasis, may also be present (Figure 4). A lung biopsy is not generally necessary, as the HRCT pattern is able to make a good prediction of the underlying histopathology [59,60,61]. Reversal of HRCT modifications is rarely achieved, and GGO is commonly associated with irreversible disease [59,60,61]. Indeed, as an improvement in HRCT findings has been observed in only 5% of patients with GGO and nonfibrotic interstitial opacities, GGO may represent fibrosis in many SSc cases [59,60,61]. Over time, the radiographic progression involves the replacement of GGO with honeycombing/traction bronchiectasis and/or bronchiolectasis [59,60,61].

However, HRTC does have some drawbacks involving the resolution limitations of CT, as anatomical structures are disclosed by variations in density measured in spatial units (‘voxels’). When intralobular fibrosis is finer than the width of the individual voxels, it does not manifest as a reticular abnormality, but rather as a diffuse increase in average density, making it impossible to distinguish from inflammatory ground glass. The likelihood of irreversible disease increases when there is an admixed reticular pattern, as first described in a study of lung disease in SSc, and when there is traction bronchiectasis [59,60,61,62,63]. Recent concern has been raised as to X-ray exposure in SSc patients, leading to the evaluation of a 9-slice HRCT protocol to reduce the radiation dose. This procedure has been demonstrated to have an accuracy of over 90%, compared to the standard HRCT [63]. It has been proposed that Quantitative CT densitometry could provide a sensitive assessment of lung structure for the monitoring parenchymal damage and that it has a good correlation with PFT [63,64,65]. However, the widespread use of the phrase ‘an alveolitis on CT’, denoting likely reversibility, is seriously problematic. Indeed, the limitations of CT on this issue have been emphasized by longitudinal studies on CT changes with and without treatment [65]. When there are associated reticular abnormalities, which are a common finding in most SSc cases, short-term disease regression is observed in only a minority of patients and, in the longer term, ground glass usually progresses to overt fibrotic change [61,62,63,64,65,66]. A serial study of 41 SSc patients reported the presence of ground glass in two-thirds of the cases but regression was observed in only 5% of them over the following 2 years, which was, moreover, often resistant to treatment [65].

Considering the increase in the number of patients with interstitial lung diseases that risk developing lung cancer, particular attention should be paid to detect and characterize any new parenchymal nodule [67].

## 6. Nonpulmonary HRCT Findings in SSc-ILD

Pulmonary hypertension, which occurs in about 10 to 12% of SSc patients, is the result of the remodeling of pulmonary arterioles that leads to a progressive increase in pulmonary vascular resistance [68] and, along with interstitial lung involvement, is the most common cardiopulmonary finding in SSc [69]. As this is a significant cause of SSc-related morbidity and mortality, any sign of pulmonary arterial hypertension on HRCT, e.g., a main pulmonary artery diameter of >29 mm, must be noted [67,68]. Although the chronic interstitial involvement of the lungs may lead to mediastinal lymph node enlargement, it may also be secondary to aspirations related to the gastric reflux often present in these patients. Pleural effusion may occur, although it is present in less than 10% of cases 70. The gastrointestinal tract is involved in up to 90% of SSc patients. Moreover, oesophageal involvement is an early sign of the disease diagnosed by a dilated esophagus on CT, with a lumen ranging from 1.2 to 4 cm, containing gas or fluid [70,71].

**Figure 5:**
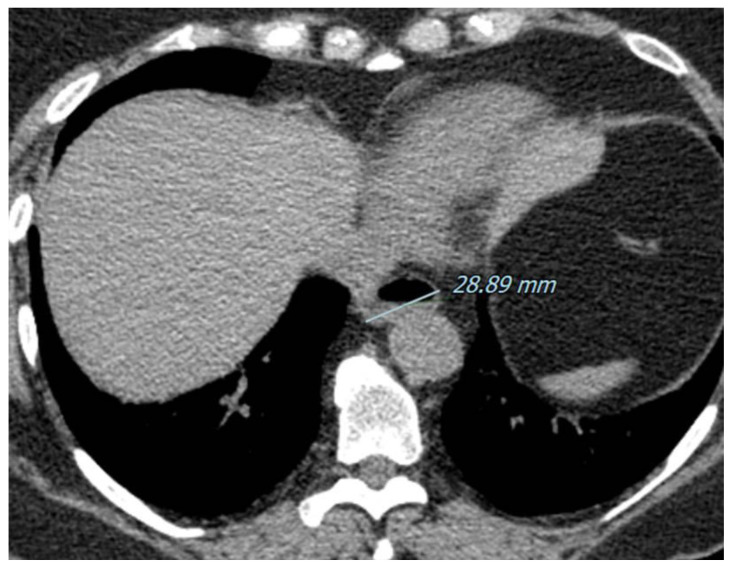
Esophageal involvement in a 42-year-old female with a diagnosis of SSc. Axial image with a mediastinal window setting shows a dilatation of the esophagus (diameter >1.2 cm) with an air-fluid level.

However, the HRCT findings in SSc are nonspecific. Indeed, there is a wide range of possible differential diagnosis, including idiopathic pulmonary fibrosis, other connective tissue diseases, drug-induced toxicity and/or asbestosis. The differential diagnosis depends on the disease stage (early vs. late) and the presence of additional findings, e.g., oesophageal involvement (Figure 5), that can be help in differentiating connective tissue related-interstitial lung diseases from other aetiologies [46] (Table 1).

## 7. CT and Radiation Risk

Radiation exposure is a potentially limiting factor for the use of HRCT [72]. Background radiation is all around us and varies from place to place and over time, depending on various factors, such as the amount of naturally-occurring radioactive elements in soil, water and air, altitude/latitude and Radon exposure. Indeed, on average, 80% of the annual dose of background radiation a person receives comes from naturally occurring terrestrial and cosmic radiation sources and is about 3 to 6 mSv. CT scans add a further dose. Moreover, whilst background radiation is spread out over time and the whole body, giving the DNA time to repair itself, a CT dose is delivered in five seconds to a specific area [72,73,74,75].

In medical practice, a patient’s exposure to radiation must be kept as low as possible. Over the last few years, focus has been on the cumulative radiation dose, a highly relevant issue when discussing the harm of CT-based screening, e.g., for lung cancer. The radiation risk at high doses of >50 mSv has been well established from atomic bomb data and an increasing number of clinical studies. It is known that the risk for lung cancer induction is higher than the risk of induction of any other cancer in the age group screened (>50 years) [72]. However, only estimates can be made in the range between 5 and 50 mSv, and a linear extrapolation to 0 is usually performed. Using this linear no threshold theory, the life-time attributable cancer risk at the age of 60, with an exposure to 1 mSv is calculated to be 1 in 20,000 [75,76,77]. The National Lung Screening Trial (NLST) estimated that approximately 8 mSv per participant was delivered over a 3-year period. Using the NLST data, these models predict that approximately one cancer death may be caused by radiation from imaging per 2500 persons screened. Another paper by Brenner et al. suggests that a single baseline CT screening examination for lung cancer using a low dose protocol represents a fairly low risk [78]. Moreover, recent findings highlight that the number of HRCT slices required can be reduced, limiting the patient’s radiation dose without affecting the lung fibrosis assessment (severity or extent), compared to standard protocols [65,66]. This approach may be particularly useful in patients with established ILD who often have regular HRCT progression monitoring. Indeed, it has been reported that sequential reduced 9-slice HRCT is able to reliably detect mild ILD and discriminate lung fibrosis at a threshold of 20%, with high sensitivity and specificity [65,66,67,68,69,70,71,72,73]. Therefore, it may well be useful in detecting early disease progression in SSc patients, although this hypothesis requires further validation in additional SSc patient cohorts. Quantitative imaging is emerging as a powerful tool to evaluate the extent of lung fibrosis and disease progression as it is able to provide more objective information than visual assessment [72,73,74,75,76,77,78,79]. This method involves the use of computer-generated algorithms to quantify the extent of features such as fibrosis, ground-glass opacities and/or honeycombing, in a uniform and standard manner and is currently being used primarily in SSc-ILD clinical trials but may well become established in daily clinical practice in due course [79,80,81]. Semiquantitative imaging may also be used for the diagnosis and quantification of lung involvement in SSc-ILD, even if this method does not eliminate assessor subjectivity or variability [80,81].

## 8. Semiquantitative Visual Scoring System

There is increasing evidence that the extent of lung fibrosis on CT is an important prognostic factor in fibrotic lung diseases [82,83,84,85,86,87,88,89,90]. In fact, a multicenter study on patients with idiopathic pulmonary fibrosis enrolled in a treatment trial reported that the CT extent of lung disease was the strongest independent predictor of survival (*p*-value= 0.0001) [82,83]. Similarly, in the control arm of a recently published Scleroderma Lung Study, the CT extent of lung fibrosis at baseline was a strong predictor of FVC at 12 months (adjusted for baseline FVC) (*p*-value= 0.006) [82,83,84,85]. A semiquantitative visual scoring system was used to evaluate the extent of disease on CT in both of these studies. However, semiquantitative scoring systems are limited by the need for expert radiologists and moderate interobserver variation. CT has been used to accurately quantify emphysema for almost two decades now, although the development of quantitative CT-based measures for lung fibrosis has been more challenging [86,87].

CT attenuation in the lung parenchyma, measured in Hounsfield units, is determined by the relative amount of air, soft tissue and blood in each volume element (voxel). The CT attenuation histogram of normal lung deviates from the Gaussian normal distribution [88,89,90,91,92,93,94]. Lung fibrosis or inflammation leads to an increase in the amount of soft tissue in the lung, it increases the average lung attenuation and decreases the sharpness of the histogram peak (kurtosis), as well as the degree of leftward skewness of the curve [88,89,90]. Hartley et al.’s study on 24 subjects with idiopathic pulmonary fibrosis (IPF) and 60 subjects with asbestosis reported that the average and median lung attenuation, derived from HRCT images, were independently associated with the presence of moderate-to-severe dyspnea, a higher profusion of chest radiograph abnormalities, a lower FVC and fewer abnormalities on BAL [89]. These data were confirmed in a more recent study on 144 IPF patients [90]. The article by Camiciottoli et al. offers a further assessment of CT histogram-based measures (average lung attenuation, skewness and kurtosis) in 48 patients with systemic sclerosis (33 with limited disease and 15 with diffuse disease), carefully characterized both by physiology and the quality-of-life scores [87]. They demonstrated that the quantitative CT-based measures are much more reproducible than semiquantitative visual scores. Furthermore, there was a statistically significant correlation between the quantitative measurements and numerous components of the quality-of-life scores, as well as with the six-minute walk test. These results provide further evidence that quantitative CT measurements offer a robust and valid assessment of the severity of lung parenchymal abnormality in patients with lung fibrosis. Several authors emphasize that CT histogram-based measurements have two potential limitations. First, they depend on the level of inspiration achieved for the scan [90,91,92,93,94]. Second, these evaluations (e.g., pulmonary physiologic tests) provide a global measurement of the normal and abnormal lung and do not directly evaluate the type or extent of abnormality [94,95]. These texture-based methods may soon allow us to reproducibly distinguish the abnormal from normal lung, providing an objective index of disease pattern and extent. Once validated, these techniques will most likely be valuable in the evaluation of treatment efficacy for fibrotic lung diseases [94,95].

## 9. The Role of HRTC and Other Imaging Techniques

In the past few years, several authors have supported the role of lung ultrasound (LUS) as a noninvasive and nonionizing imaging method for detecting ILD [96]. Discrete vertical hyperechoic reverberation artifacts arising from the pleural line and extending to the bottom of the screen without fading when the lung parenchyma air content is decreased or when the interstitial space is expanded, known as B-lines, have been observed in 51% of the patients with SSc [97]. Several studies have reported a significant linear correlation between the number of B-lines and HRCT score [88,89,90,91,92,93,94,95,96,97,98,99,100,101]. Ultrasound may also represent a useful tool for the detection of ILD at an early stage in patients with SSc [98,99,100,101,102].

Further imaging techniques with potential application in early SSc-ILD, particularly magnetic resonance imaging (MRI), have been proposed [103,104]. However, in comparison with HRCT, MRI was found to be less sensitive and under-measured disease extent [104,105]. In conclusion, at present these methods are investigational in relation to the diagnosis and assessment of SSc-ILD and not commonly used currently in clinical practice [103,104,105].

## 10. Conclusions

Considering the heterogeneity of SSc-ILD, the potential absence of symptoms in early or mild disease, lung function tests and chest imaging can help to identify who has SSc-associated ILD and predict progression [1,2,3]. Furthermore, patients may be diagnosed earlier and more efficiently through multidisciplinary evaluation and collaboration, an essential element to optimize and improve clinical outcomes [1,2,106,107,108,109]. Several studies have suggested that all SSc patients should undergo a baseline pulmonary function test and lung HRCT screening to allow for an early diagnosis of ILD and tailor further management. Indeed, a subset of patients with SSc-ILD develop progressive ILD, which is associated with a higher mortality, even if the prevalence of progressive ILD and the overall disease course and patterns of SSc-ILD are still a question of debate [1,2,3,4,5]. However, there is general agreement that early treatment is required to prevent progression aimed at avoiding irreversible organ damage [1,2,3]. Most international literature, in agreement with our experience, emphasises the heterogeneity and variability of the course of ILD in SSc and underlines the need for careful monitoring of all patients with SSc-ILD by both lung function tests and HRTC [1,2,3,4,5].

## Figures and Tables

**Table 1:** Main HRCT differential diagnoses of systemic sclerosis.

Early stage	NSIP pattern: Connective tissue diseases, other than systemic sclerosis (e.g., rheumatoid arthritis, polymyositis/dermatomyositis, Sjögren’s syndrome, antisynthetase syndrome) Drug-associated NSIP (methotrexate, amiodarone, chemotherapeutic agents, nitrofurantoin) Interstitial pneumonia with autoimmune features Hypersensitivity pneumonitis Idiopathic NSIP
Late fibrotic stage	Fibrotic NSIP/UIP pattern: Connective tissue diseases, other than systemic sclerosis (e.g., rheumatoid arthritis) Drug toxicity (e.g., bleomycin) Chronic hypersensitivity pneumonitis Asbestosis Idiopathic pulmonary fibrosis

## Data Availability

Not applicable, this is a Review.
